# Orf virus induces complete autophagy to promote viral replication via inhibition of AKT/mTOR and activation of the ERK1/2/mTOR signalling pathway in OFTu cells

**DOI:** 10.1186/s13567-023-01153-1

**Published:** 2023-03-14

**Authors:** Lijun Lv, Jiyu Guan, Ruixue Zhen, Pin Lv, Mengshi Xu, Xingyuan Liu, Shishi He, Ziyu Fang, Zi Li, Yungang Lan, Huijun Lu, Wenqi He, Feng Gao, Kui Zhao

**Affiliations:** 1 Key Laboratory of Zoonosis, Ministry of Education, College of Veterinary Medicine, Jilin University, Changchun, China; 2 Key Laboratory of Zoonosis, Ministry of Education, Institute of Zoonosis, Jilin University, Changchun, China

**Keywords:** ORFV, autophagy, PI3K/AKT/mTOR, ERK1/2/mTOR, replication

## Abstract

Orf virus (ORFV) is the causative agent of contagious ecthyma, which is an important zoonotic pathogen with a widespread distribution affecting sheep, goats and humans. Our previous research showed that autophagy can be induced in host cells by ORFV infection. However, the exact mechanism of ORFV-induced autophagy remains unknown. In this study, we investigated the underlying mechanisms of autophagy induced by ORFV in OFTu cells and the impact of autophagy on ORFV replication. By using specific autophagy inhibitors and activators, Western blotting, immunofluorescence and transmission electron microscopy imaging, we confirmed that ORFV infection triggered intracellular autophagosome accumulation and the activation of autophagic flux. Moreover, ORFV-induced autophagic activity was found to rely on an increase in the phosphorylation of tuberous sclerosis complex 2 (TSC2) and a decrease in the phosphorylation of mammalian target of rapamycin (mTOR), which is mediated by the suppression of the PI3K/AKT/mTOR signalling pathway and activation of the ERK1/2/mTOR signalling pathway. Furthermore, we investigated the role of mTOR-mediated autophagy during ORFV replication using pharmacological agents and demonstrated that ORFV-induced autophagy correlated positively with viral replication. Taken together, our data reveal the pathways of ORFV-induced autophagy and the impact of autophagy on ORFV replication, providing new insights into ORFV pathogenesis.

**Supplementary Information:**

The online version contains supplementary material available at 10.1186/s13567-023-01153-1.

## Introduction

Orf is also called contagious ecthyma or contagious pustular dermatitis, which is a zoonotic infectious disease caused by Orf virus (ORFV). ORFV belongs to the *Parapoxvirus* genus in the *Poxviridae* family and affects an increasing number of host species, including but not limited to sheep and goat [1]. The disease caused by ORFV is characterized by ulcerating lesions on the skin and mucous membranes of the mouth, lips and nose of the affected animals that usually resolve spontaneously over time [2]. As a linear double-stranded DNA virus, ORFV can reinfect a host [3], to some extent, which is related to the development of a range of virulence and immunomodulatory factors, including ovine interferon resistance protein (OVIFNR), granulocyte/macrophage colony-stimulating factor (GM-CSF) inhibitory factor (GIF), viral vascular endothelial growth factor-E (VEGF-E) and the viral orthologue of mammalian IL-10 (vIL-10) [4].

Macroautophagy (hereafter referred to as autophagy) is a highly conserved catabolic process that can maintain cellular homeostasis by recovering/recycling cellular components (such as protein, glycogen, lipids, DNA, and RNA) and organelles (such as the endoplasmic reticulum, mitochondria and ribosomes) [5] or then transporting them to lysosomes, where they are degraded [6]. The regulatory molecular mechanisms underlying mammalian autophagy can be subdivided into four different stages: autophagosome formation initiation, nucleation, maturation, fusion and degradation [7]. Autophagy is regulated by autophagy-related proteins (ATGs), such as the unc-51-like autophagy-activating kinase (ULK) 1/2 complex, Beclin1 (BECN1), microtubule-associated protein 1 light chain 3B (LC3B) [8], and other nonautophagy-related proteins [e.g., mammalian target of rapamycin (mTOR), the kinases AMP-activated protein kinase (AMPK), extracellular signal-regulated kinase 1/2 (ERK1/2) and serine/threonine kinase (AKT)] [7, 9]. Autophagy and the related pathways have been reported to play crucial roles in many processes involving nutrient starvation, oxidative stress, cancer, ageing, pathogenic infection and other diseases [10–14].

Autophagy plays dual roles in response to pathogen infection. Autophagy-mediated innate immunity is a critical antiviral defence mechanism; however, many viruses have evolved the ability to inhibit and circumvent autophagy-induced viral degradation to benefit their own survival [15]. The role of autophagy in antiviral immunity has been well characterized. Some viruses, such as Peste des petits ruminants virus (PPRV) [16], hepatitis delta virus (HDV) [17], Junín virus (JUNV) [18] and porcine epidemic diarrhoea virus (PEDV) [19], have been reported to induce autophagy in different ways. To counteract autophagy-mediated antiviral defence mechanisms, a variety of viruses, including pseudorabies virus (PRV) [20], African swine fever virus (ASFV) [21], Epstein‒Barr virus (EBV) [22] and SARS-CoV-2 [23], can inhibit autophagy by regulating a key step in the autophagic pathway. Although in our previous study we reported that ORFV induces autophagy, the regulatory mechanisms involved in ORFV-induced autophagy and the mechanism underlying the role of autophagy in ORFV replication remain unclear.

In the present study, we demonstrated that ORFV induces complete autophagy via the activation of specific inhibitors and activators by performing Western blotting, immunofluorescence assays and transmission electron microscopy imaging. Furthermore, ORFV has been suggested to regulate host pathways that increase autophagy, and we confirmed that it exerts its regulatory effects by suppressing the PI3K/AKT/mTOR signalling pathway and activating the ERK1/2/mTOR signalling pathway. Additionally, we investigated the role of autophagy in ORFV replication using pharmacological agents and demonstrated that ORFV-induced autophagy promoted viral replication. In conclusion, our results indicate that ORFV induces complete autophagy to promote viral replication mainly by targeting mTOR pathways. Hence, the study increases the understanding of ORFV pathogenesis and provides new insights into the development of effective therapeutic strategies.

## Materials and methods

### Cells and viruses

Primary ovine foetal turbinate (OFTu) cells were cultured in Dulbecco’s modified Eagle medium (DMEM) (Gibco, Grand Island, NY) containing 10% foetal bovine serum (FBS) (BioInd, Kibbutz Beit, Israel) and a 1% penicillin‒streptomycin solution and were maintained in DMEM containing 2% FBS in a humidified CO_2_ incubator at 37 °C. The ORFV-CL18 strain (GenBank accession number MN648219) used in this study was isolated, identified in our laboratory and propagated in OFTu cells.

### Chemical treatment

Chemical activators and inhibitors were dissolved in DMSO (Sigma, St. Louis, MO, USA) or sterile water at a high concentration for storage as described in the instructions of the chemical manufacturers. Then, they were diluted with fresh DMEM with or without FBS before use. The reagents used in this study included 15 μM and 30 μM rapamycin (Selleck, Houston, TX, USA), 2.5 mM and 5 mM 3-methyladenine (Selleck), 0.5 nM and 1 nM bafilomycin A1 (Selleck), 15 μM and 30 μM SC79 (Meilunbio, Shanghai, China), 10 μM and 20 μM U0126 (Meilunbio), 1.5 mM and 3 mM 5-aminoimidazole-4-carboxamide ribonucleoside (AICAR; Meilunbio), 0.5 mM and 1 mM Compound C (Meilunbio), and DMSO.

OFTu cells were treated with autophagy activators (rapamycin) or inhibitors (3-methyladenine, bafilomycin A1), an AKT activator (SC79), an inhibitor of ERK signalling pathway (U0126), and an AMPK activator (AICAR) and inhibitor (Compound C). Briefly, OFTu cells were rinsed with PBS when they reached approximately 70% confluency. Subsequently, different concentrations of chemical reagents were added to each well containing cells, and the cells were incubated at 37 °C in a 5% CO_2_ incubator for 1 h. Then, the medium was replaced with fresh DMEM, and the OFTu cells were infected with ORFV (MOI = 10). After absorption was allowed to proceed for 1 h, the supernatant was removed, and the OFTu cells were treated with chemicals to determine the half-maximal effective concentration and incubated at 37 °C in a 5% CO_2_ incubator for the indicated times. For establishing vehicle controls, DMSO at the same concentration as the chemical agent was added to the culture medium.

### Plasmid construction and transfection

Full-length ovine Rheb (238566765) was amplified from OFTu cell cDNA and cloned into a PCMV-C-Flag vector (Beyotime Biotech, Shanghai, China) by using a Basic Seamless Cloning and Assembly Kit (TransGen Biotech, Beijing, China). After sequencing by Sangon Biotech Company (Shanghai, China), the PCMV-C-Flag-Rheb plasmid was mutated using a Fast Mutagenesis Kit (Vazyme Biotech, Nanjing, China). After the glutamine at amino acid position 64 in Rheb was replaced with a leucine residue, the eukaryotic expression vector PCMV-C-Flag-RhebQ64L, which showed the ability to activate mTOR [24], was constructed. A full-length ovine AKT (240849341) was amplified from OFTu cell cDNA and cloned into a PCMV-Myc-N vector (Beyotime Biotech, Shanghai, China) by using a Basic Seamless Cloning and Assembly kit (TransGen Biotech). The PCMV-Myc-N-AKT plasmid was shown to have been constructed successfully by sequencing. The primers used for PCR and PCR point mutations are listed in Table 1.

**Table 1:** **PCR primers used in the study**

Primers	Sequence (5′-3′)
PCMV-C-Flag-Rheb-F	CCAAGCTTCTGCAGGAATTCATGCCGCAGTCCAAGTCC
PCMV-C-Flag-Rheb-R	TTGTAATCTCTAGACTCGAGCATCACCGAGCAGGAAGACT
PCMV-C-Flag-RhebQ64L-F	AGCTGGGCTGGATGAATATTCCATCTTTCCTCAG
PCMV-C-Flag-RhebQ64L-R	ATTCATCCAGCCCAGCTGTGTCCACAAGTTGA
PCMV-Myc-N-Akt-F	GGCCATGGAGGCCCGAATTCGGATGAACGACGTGGCCGT
PCMV-Myc-N-Akt-R	GCGGCCGCGGTACCTCAGGCCGTGCCGCTG

When the confluency was approximately 70%, the OFTu cells were rinsed with PBS and then transfected with the above recombinant plasmids using Lipofectamine 3000 reagent (Invitrogen, Carlsbad, CA). Briefly, OFTu cells were covered with Opti-MEM (Gibco) before transfection, and plasmids were mixed with Lipofectamine 3000 reagent and incubated as described in the instructions. After incubation for 6 h at 37 °C, the medium was removed, and the OFTu cells were grown in fresh DMEM with 2% FBS and treated for the indicated times.

### Western blotting analysis

OFTu cells were infected with ORFV (MOI = 10) for 36–60 h or treated with chemical activators or inhibitors and then infected with ORFV (MOI = 10) for 60 h and harvested. Cell lysates were prepared by using RIPA lysis buffer (Beyotime) with 1 mM PMSF and quantified with a Pierce BCA protein assay kit (Thermo, Marina, CA, USA). Total protein was resolved by sodium dodecyl sulfate‒polyacrylamide gel electrophoresis (SDS‒PAGE) and then transferred to a polyvinylidene difluoride membrane (Millipore, Billerica, MA, USA). The membrane was blocked with blot-blocking buffer (NCM Biotech, Suzhou, China) and incubated with primary antibodies. Antibodies against the following proteins were used in this study: LC3B (Sigma, St. Louis, MO, USA), SQSTM1/P62 (CST, Beverly, MA, USA), phosphorylated and nonphosphorylated mTOR, TSC2, AMPK, AKT, PI3K, ERK1/2, p70S6K (CST), Rheb and GAPDH. In addition HRP-conjugated AffiniPure goat anti-rabbit IgG and HRP-conjugated AffiniPure goat anti-mouse IgG (Proteintech Group, Inc. Rosemont, IL, USA) were used. The blots were visualized with an enhanced ECL chemiluminescent substrate (Biosharp, Beijing, China) in a chemiluminescence imaging system (Tanon, Shanghai, China) after being washed and incubated with secondary antibody, and the expression of the proteins was quantified using ImageJ.

### Confocal laser scanning microscopy

OFTu cells were grown on sterile glass slides (20 mm in diameter), which were placed into every well of a 12-well plate. After 24 h, green fluorescence protein conjugated with microtubule-associated protein 1 light chain 3 (GFP-LC3) and mRFP-GFP-LC3 plasmids constructed in our laboratory were transfected as described above. Twenty-four hours post-transfection, the cells were infected with ORFV (MOI = 10) or treated with 30 μM rapamycin for the indicated times, and uninfected cells were used as negative controls.

Then, 36–60 h post-infection (hpi), OFTu cells were washed twice with PBS, fixed with 4% paraformaldehyde at room temperature for 30 min, and incubated with Hoechst 33258 (Beyotime Biotech) at room temperature for 15 min. Then, glass slides were mounted with antifade mounting medium (Beyotime Biotech) and removed from the wells. Fluorescence images were captured via confocal laser scanning microscopy.

### Transmission electron microscopy

OFTu cells infected with ORFV (MOI = 10) for 36–60 h were harvested and fixed with 2.5% glutaraldehyde solution and 1% osmic acid. Then, the cells were dehydrated in different concentrations of ethanol and acetone solution and embedded in epoxy resin at 37 °C for 3 h. Ultrathin sections were prepared and stained with both uranyl acetate and lead citrate and then were examined with a transmission electron microscope.

### CCK8 assay

A CCK8 assay was performed to measure the effect of chemical activators or inhibitors on cell proliferation. OFTu cells were subcultured to a 96-well plate, and fresh DMEM containing 2% FBS with different concentrations of chemicals was added when cells reached approximately 70% confluency. After treatment for 60 h, the medium was removed, and the cells were rinsed twice with PBS at pH 7.4. Then, DMEM without FBS or penicillin‒streptomycin solution was added to each well. Finally, the CCK8 reagent in medium at a of 1:10 was added to each well, and the cells were incubated for 3 h. The optical density of each well was read at 450 nm using a microplate reader (Bio-Rad, Hercules, CA, USA).

### Viral titre assay

OFTu cells were seeded into T25 cell culture flasks and pretreated with 30 μM rapamycin, 5 mM 3-methyladenine or 1 nM bafilomycin A1 diluted with fresh DMEM for 1 h when the cells reached approximately 80% confluency. The cells were rinsed with PBS after removing the supernatant and then infected with ORFV. After incubation for another 1 h, the cells in each flask were rinsed with PBS and treated with 15 μM rapamycin, 2.5 mM 3-methyladenine or 0.5 nM bafilomycin A1 diluted in fresh DMEM containing 2% FBS. After incubation for 24–36 h at 37 °C, the cytopathic effect (CPE) was measured, and the cells seeded into T25 cell culture flasks were harvested 60 hpi, frozen at −80 °C and thawed three times at 4 °C. The supernatants were harvested, and the viral titres were determined by TCID_50_ assay. The viral titre (TCID_50_/0.1 mL) was calculated via the Reed–Muench method.

### Plaque assay

When autophagy was pharmacologically induced by rapamycin or inhibited by 3-methyladenine and bafilomycin A1, a virus plaque assay was performed to determine the effect of autophagy on ORFV replication. The procedure was performed as follows: ORFV was diluted tenfold with fresh DMEM until the final ORFV concentrations reached 10^–2^ and 10^–3^. When OFTu cells reached approximately 90% confluency, 300 μL of diluted ORFV was added to each well, with three wells established for each dilution, and then incubated at 37 °C in a 5% CO_2_ incubator. After incubation for 1 h, 1.5% low-melting-point agarose (Solarbio) preheated in a 42 °C water bath was mixed with 2 × MEM (Gibco, Grand Island, NY, USA) at a ratio of 1:1, and 2% FBS and 1% penicillin‒streptomycin solution were added to the agarose-MEM mixture. Then, this solution was added to each well of a 24-well plate. The 24-well plate was inverted when the solution solidified and was incubated at 37 °C in a 5% CO_2_ incubator for 5–7 days. After an obvious CPE was observed, the cells were fixed with 5% formaldehyde solution for 10 min and stained with crystal violet (Solarbio) for 15 min. Finally, the OFTu cells were washed with ddH2O, and the viral plaques were observed by the naked eye. The virus titres (PFU/mL) were calculated on the basis of the following formula: PFU/mL = A × B^−1^ × C^−1^ (A: number of plaques; B: dilution factor; and C: inoculum volume (mL)).

### Statistical analysis

Statistical analysis was performed with unpaired t tests and one-way ANOVA for multiple comparisons using GraphPad Prism 7.0. All the data were obtained from at least three independent experiments and are presented as the mean ± standard deviation (SD). A *P* value < 0.05 was considered to be statistically significant (*P < 0.05, ** P < 0.01, *** P < 0.001 and **** P < 0.0001), and “ns” indicates a difference that was not significant.

## Results

### ORFV-CL18 infection increases autophagy in OFTu cells

**Figure 1:**
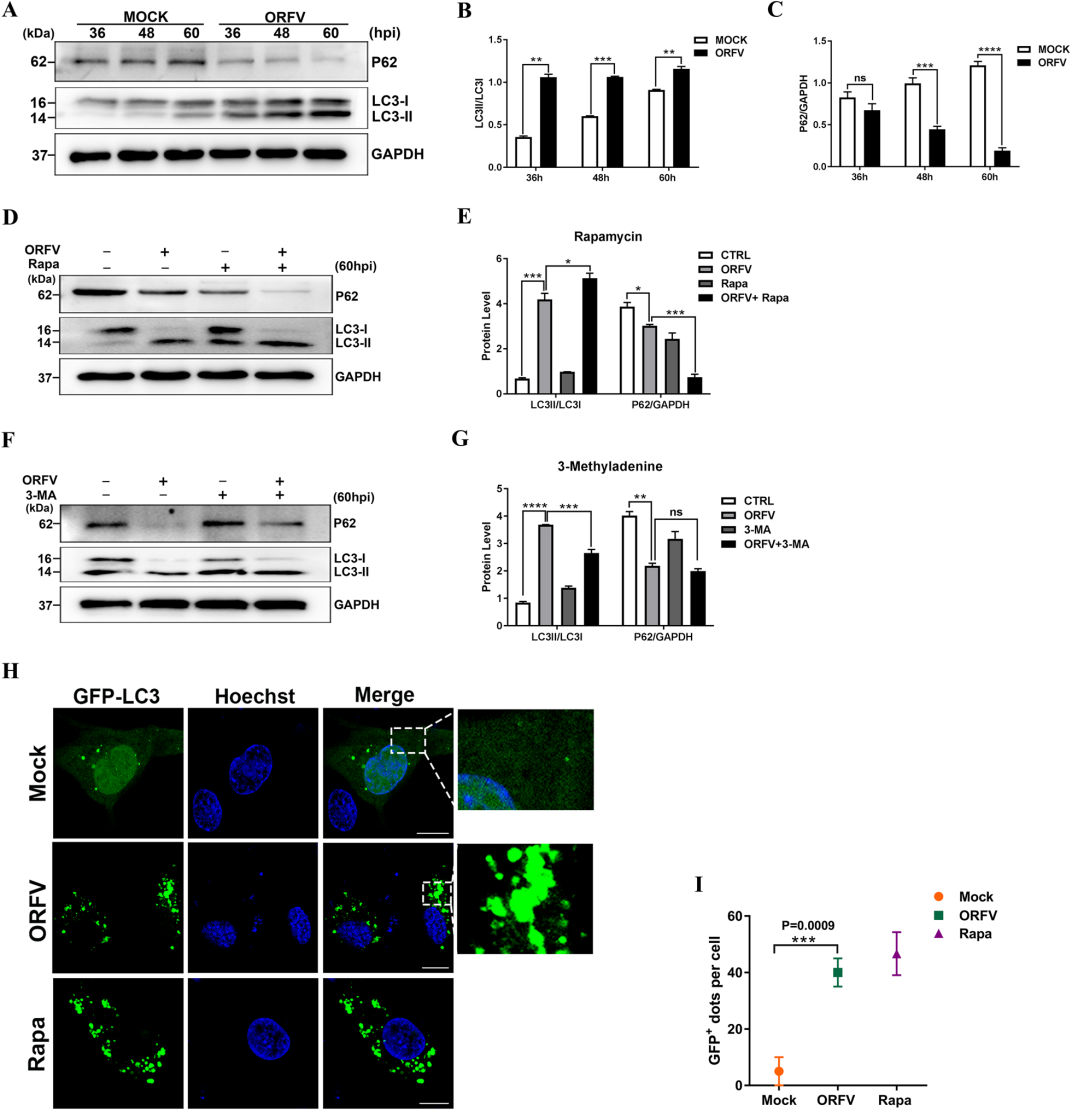
**Autophagy is induced in ORFV-infected OFTu cells. A** OFTu cells were mock infected or infected with ORFV-CL18 (MOI = 10), and 36, 48 and 60 hpi, the expression levels of the autophagy marker proteins LC3B and P62 were measured by Western blotting. GAPDH was used as the loading control. **B**, **C** The ratios of LC3-II to LC3-I **B** and P62 to GAPDH **C** were quantified by ImageJ software. The results were expressed as the mean ± SD (*n* = 3) (**P < 0.01, ***P < 0.001, ****P < 0.0001; unpaired t test). **D** OFTu cells were mock infected, infected with ORFV-CL18, treated with 30 μM rapamycin (Rapa) or treated with 30 μM Rapa, infected with ORFV and then cultured for 60 h. The expression levels of LC3B and P62 were measured by Western blotting. GAPDH was used as the loading control. **E** The ratios of LC3-II to LC3-I and P62 to GAPDH after different treatments. The results are expressed as the mean ± SD (*n* = 3) (*P < 0.05, *** P < 0.001; one-way ANOVA). **F** OFTu cells were mock infected, infected with ORFV-CL18, treated with 5 mM 3-methyladenine (3-MA) or treated with 5 mM 3-MA, infected with ORFV and cultured for 60 h. The expression levels of LC3B and P62 were measured by Western blotting. GAPDH was used as the loading control. **G** The ratios of LC3-II to LC3-I and P62 to GAPDH after different treatments. The results are expressed as the mean ± SD (*n* = 3) (**P < 0.01, *** P < 0.001, **** P < 0.0001; one-way ANOVA). **H** OFTu cells were mock infected, infected with ORFV-CL18 or treated with 30 μM Rapa after transfection with the GFP-LC3 vector and cultured for 24 h. Autophagosomes were observed by confocal laser scanning microscopy. Scale bar, 10 μm. **I** Fluorescence images were analysed with ImageJ software, and GFP-positive puncta were quantified, and the results are expressed as the mean ± SD (*n* = 3) (***P < 0.001; unpaired t test).

Microtubule-associated protein light-chain 3 (LC3) and P62 are often used as autophagy markers. By monitoring the expression levels of LC3B and P62, we found that autophagy was induced in OFTu cells infected with ORFV-CL18. As shown in Figures 1A–C, the conversion of LC3-I to LC3-II was obvious, and the expression level of P62 was significantly downregulated in OFTu cells infected with ORFV-CL18. To confirm these findings, the autophagy activator rapamycin was used as a positive control to activate autophagy because it specifically acts as an allosteric inhibitor of mTORC1. Compared with normal cells, the conversion of LC3-I to LC3-II increased (the ratio of LC3-II to LC3-I was increased) in OFTu cells treated with rapamycin; however, the expression level of P62 was reduced, and it was expressed at a level similar to that in ORFV-infected cells (Figures 1D, E). In addition, as a widely used autophagy inhibitor because of its inhibitory effect on class III PI3K, 3-methyladenine was used as the negative control. In contrast to the rapamycin treatment, 3-methyladenine treatment did not significantly induce the conversion of LC3-I to LC3-II, and the expression of P62 was not significantly changed, in contrast to the effect on virus-infected cells (Figures 1F, G). Additionally, ORFV-infected OFTu cells were transfected with a GFP-LC3 expression vector, which enabled the quantification of autophagosomes, which is an indicator of autophagy. Under normal conditions, the GFP-LC3 fusion protein was diffusely distributed in the cytoplasm but translocated to the autophagosome membrane when autophagy was activated. As shown in Figure 1H, GFP green fluorescence spots were observed in ORFV-infected OFTu cells via confocal microscopy, and this was the same observation as that made with rapamycin-treated cells; however, the green fluorescence was diffusely distributed throughout the cytoplasm in normal cells. These results were confirmed by fluorescence image analysis with ImageJ software, and GFP-positive spots in different treatment groups were quantified and analysed via unpaired t test. The number of GFP-positive spots in cells infected with ORFV-CL18 increased significantly compared with those in mock-infected OFTu cells, which was an effect similar to that on cells treated with Rapa (Figure 1I). Hence, we determined that ORFV induced autophagosome formation. These results indicated that ORFV infection activated autophagy.

### ORFV-CL18 induces autophagic flux and autolysosome formation

**Figure 2:**
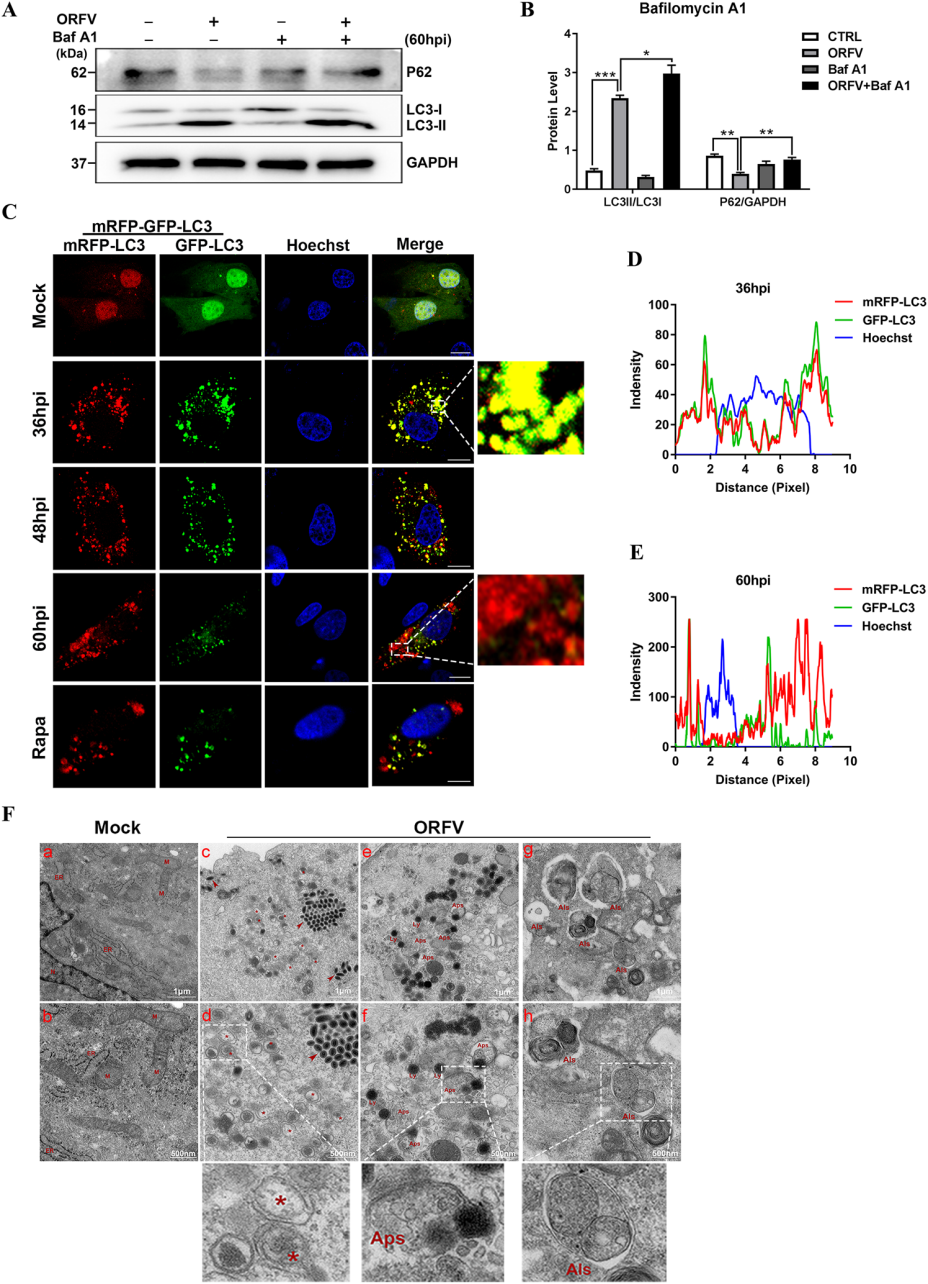
**ORFV-CL18 induces autophagic flux and the formation of autophagosomes and autolysosomes. A** OFTu cells were mock infected, infected with ORFV-CL18, treated with 1 nM bafilomycin A1 (Baf A1) or treated with 1 nM Baf A1, and then infected with ORFV (MOI = 10). At 60 hpi, the expression levels of the autophagy marker proteins LC3B and P62 were measured by Western blotting. GAPDH was used as the loading control. **B** The ratios of LC3-II to LC3-I and P62 to GAPDH in different treatment groups. LC3-II and LC3-I protein levels were quantified by ImageJ software, and the ratio of LC3-II to LC3-I is expressed as the mean ± SD (*n* = 3) (*P < 0.05, *** P < 0.001; one-way ANOVA). The p62 protein expression level was normalized to that of GAPDH using ImageJ software, and the results are expressed as the mean ± SD (*n* = 3) (**P < 0.01; one-way ANOVA). **C** Autophagosome and autolysosome formation was detected by fluorescence assay. Briefly, OFTu cells transfected with the mRFP-GFP-LC3 expression vector were mock infected, infected with ORFV-CL18 and incubated for 36 h, 48 h and 60 h or treated with 30 μM rapamycin (Rapa) and incubated for 60 h. After treatment, the formation of autophagosomes (yellow puncta) and autolysosomes (red puncta) was analysed via confocal laser scanning microscopy. Scale bar, 10 μm. **D**, **E** The fluorescence signals of mRFP-LC3 (red) and GFP-LC3 (green) were measured by ImageJ/FIJI 36 hpi **D** and 60 hpi **E**, respectively. **F** OFTu cells were mock infected or infected with ORFV-CL18. At 36, 48 and 60 hpi, the morphology of subcellular organelles was observed by transmission electron microscopy. Asterisks indicate double-membrane vesicles, and red arrows indicate ORFV virions. (*M* mitochondria, *ER* endoplasmic reticulum, *N* nuclei, *Aps* autophagosome, *Als* autolysosome, *Ly* lysosome.) (Scale bars of a, c, e and g, 1 μm; scale bars of b, d, f and h, 500 nm).

Autophagy involves dynamic and complicated processes, and the number of autophagosomes is dynamically changed. Therefore, the accumulation of LC3-II can represent either the formation of autophagosomes or the disruption of the conversion of autophagosomes to autolysosomes. In this study, bafilomycin A1 was used as an autophagy inhibitor at the late stage to block autophagosome–lysosome fusion and inhibit the acidification and protein degradation in lysosomes. As shown in Figures 2A, B, the ratio of LC3-II/LC3-I in OFTu cells infected with ORFV was increased, but the expression level of P62 was decreased. When ORFV infection was combined with bafilomycin A1 treatment, LC3-II expression was increased compared to that in the untreated ORFV-infected control group, and the expression level of P62 was also increased (Figures 2A and B), indicating that ORFV infection induced autophagosome formation. Moreover, the mRFP-GFP-LC3 expression vector was transfected to estimate the autophagic flux in OFTu cells infected with ORFV. because of the low stability of GFP in acidic environment, the green fluorescence is quenched in the acidic environment of lysosomes, but the red fluorescence emitted by RFP remains stable. Hence, an mRFP-GFP-LC3 vector can be used to trace the dynamic changes in autophagic flux via the emission of different fluorescence colours. As observed with confocal microscopy, no autophagosome structure was observed in the control group, and both red and green fluorescence were diffusely distributed throughout the cytoplasm. At 36 hpi and 48 hpi, yellow fluorescent punctate-like autophagosomes were clearly observed in OFTu cells infected with ORFV-CL18 (Figures 2C and D), which was an effect similar to that in the positive control group treated with rapamycin. Nevertheless, at 60 hpi, a substantial number of red fluorescent puncta, not yellow puncta, were observed in the cytoplasm (Figures 2C and E), and they were considered markers of autolysosome formation. These results suggested that ORFV-activated autophagy induced the formation of autophagosomes and autolysosomes. In addition, as described above (Figures 1A and C, 2A, B), the expression of P62 was significantly reduced in OFTu cells infected with ORFV, which was consistent with the presentation in Figure 2C. Because it is an autophagic receptor, P62 expression is inversely proportional to autophagy activity. Thus, a decrease in P62 expression indicated that autophagic flux had been increased. To confirm this outcome, transmission electron microscopy was used to observe the morphological changes in subcellular organelles in OFTu cells infected with ORFV. As shown in Figure 2F, in the control cells, the mitochondria and endoplasmic reticulum were clearly observed, and their morphological features were normal (Figure 2F, panels a and b). In ORFV-infected cells, many oval viral particles (red arrows) arranged in a lattice-like appearance and many c-shaped double-membrane vesicles were observed in the cytoplasm, which indicated the formation of the preautophagosomal structure (Figure 2F, panels c and d). In addition, some cells showed cytoplasmic vacuolation. Some mitochondria were swollen, with some mitochondrial cristae shortened, and the number of cristae was decreased. The endoplasmic reticulum was expanded. Notably, typical double-membrane autophagosomes (Figure 2E, panels e and f) and single-membrane autolysosomes (Figure 2F, panels g and h) were observed in cells. There were circular or oval-shaped structures of different sizes containing undegraded cytosolic components and hair-like myelin figures in the autolysosomes. (Figure 2F, panels g and h). These observations indicated that ORFV-activated autophagy, as indicated by the formation of autophagosomes and autolysosomes, in which cellular substances are degraded. In summary, ORFV induced autophagic flux and autolysosome formation.

### ORFV-CL18 induces autophagy by inhibiting PI3K/AKT/mTOR signalling

**Figure 3:**
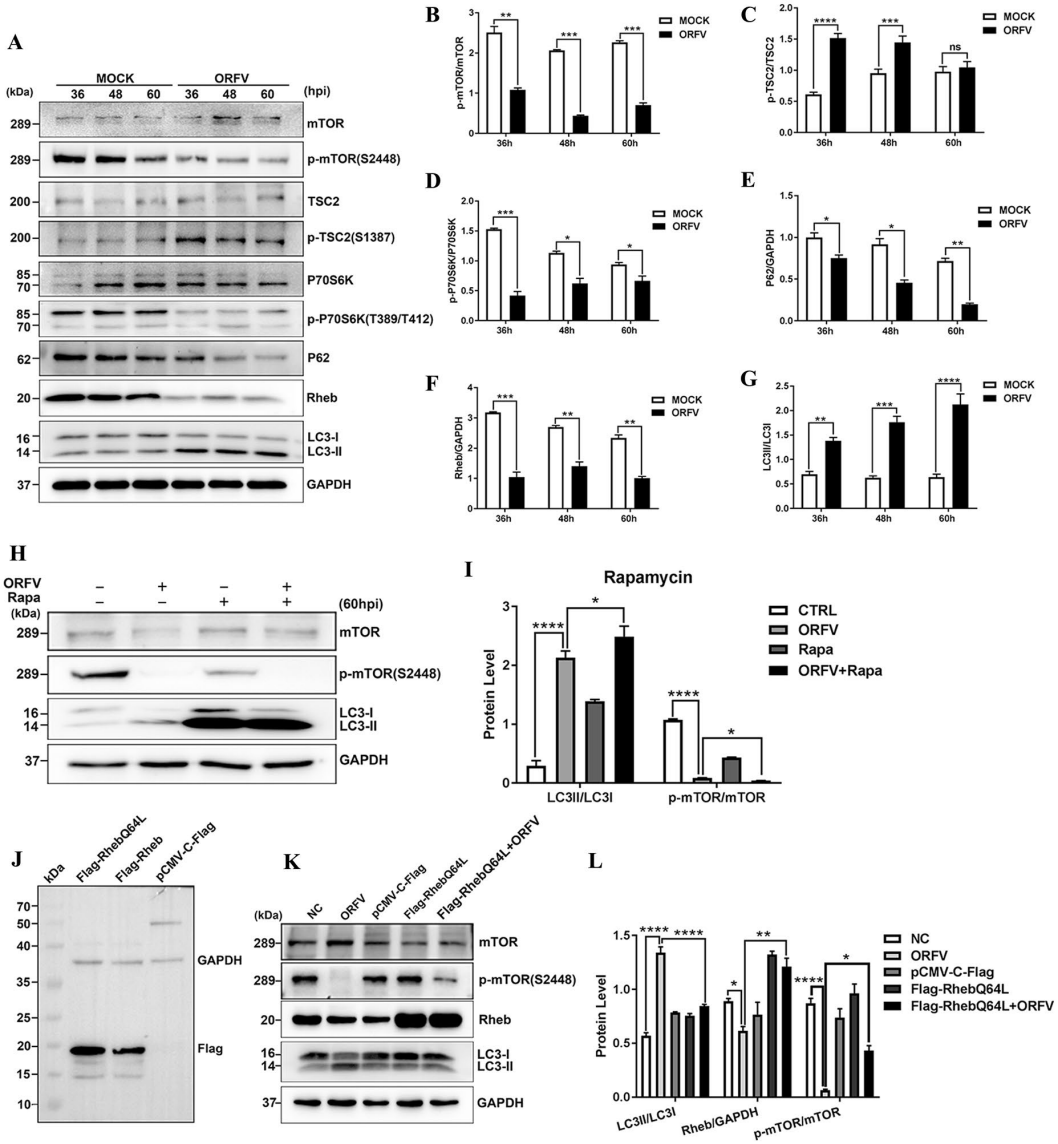
**ORFV induces autophagy by inhibiting the mTOR signalling pathway. A** OFTu cells were mock infected or infected with ORFV-CL18. At 36, 48 and 60 hpi, the expression levels of p-mTOR (S2448), mTOR, p-TSC2 (S1387), TSC2, p-P70S6K (T389/T412), P70S6K, P62, Rheb and LC3B were measured by Western blotting. GAPDH was used as the loading control. **B**–**G** The ratios of p-mTOR/mTOR **B**, p-TSC2/TSC2 **C**, p-P70S6K/P70S6K **D**, P62/GAPDH **E**, Rheb/GAPDH **F** and LC3-II/LC3-I **G**. The results are expressed as the mean ± SD (*n* = 3) (*P < 0.05, ** P < 0.01, *** P < 0.001, **** P < 0.0001; unpaired t test). **H** OFTu cells were mock infected, infected with ORFV-CL18, treated with 30 μM rapamycin (Rapa) or treated with 30 μM Rapa, infected with ORFV (MOI = 10) and cultured for 60 h. After treatment, the expression levels of p-mTOR (S2448), mTOR and LC3B were measured by Western blotting. GAPDH was used as the loading control. **I** LC3-II/LC3-I and p-mTOR/mTOR protein levels were quantified by ImageJ software, and the ratios are expressed as the mean ± SD (*n* = 3) (*P < 0.05, **** P < 0.0001; one-way ANOVA). **J** The recombinant vector pCMV-C-Flag-Rheb carrying the Rheb gene and its mutant pCMV-C-Flag-RhebQ64L were identified by Western blotting. GAPDH was used as the loading control. **K** OFTu cells were mock infected, infected with ORFV-CL18, transfected with pCMV-C-Flag or pCMV-C-Flag-RhebQ64L or transfected with pCMV-C-Flag-RhebQ64L, infected with ORFV (MOI = 10) and incubated for 60 h. After treatment, the expression levels of the autophagy marker proteins LC3B, Rheb, mTOR and p-mToR were measured by Western blotting. GAPDH was used as the loading control. **L** The ratios of LC3-II to LC3-I, Rheb to GAPDH and p-mToR to mTOR were measured, and the results are expressed as the mean ± SD (*n* = 3) (*P < 0.05, ** P < 0.01, **** P < 0.0001; one-way ANOVA).

As the main hub of autophagy regulation, mTORC1 plays a crucial role in autophagy-related signalling pathways. Therefore, we investigated whether ORFV infection induced autophagy mediated via mTOR. As a negative regulator, TSC2 was phosphorylated, the level of phosphor-TSC2 was increased, the positive regulator P70S6K was phosphorylated, the level of phosphor-P70S6K was decreased, and that of Rheb was decreased compared to the levels in uninfected control cells. Moreover, phosphor-mTOR was significantly reduced and the ratio of LC3-II/LC3-I was increased in ORFV-infected cells. (Figures 3A–G). The mTOR inhibitor rapamycin significantly reduced phosphor-mTOR level and increased the LC3-II/LC3-I ratio (Figures 3H, I), which suggested that mTOR signalling was involved in ORFV-mediated autophagy. To further determine whether mTOR signalling was essential for ORFV-induced autophagy, we constructed a mutant expression vector RhebQ64L, which constitutively activated Rheb. As shown in Figures 3K, L, the expression of RhebQ64L increased the Rheb-restored phosphor-mTOR level in ORFV-infected OFTu cells and reduced the ratio of LC3-II/LC3-I (Figures 3K, L), demonstrating that ORFV induced autophagy by inhibiting mTOR signalling in OFTu cells.

**Figure 4:**
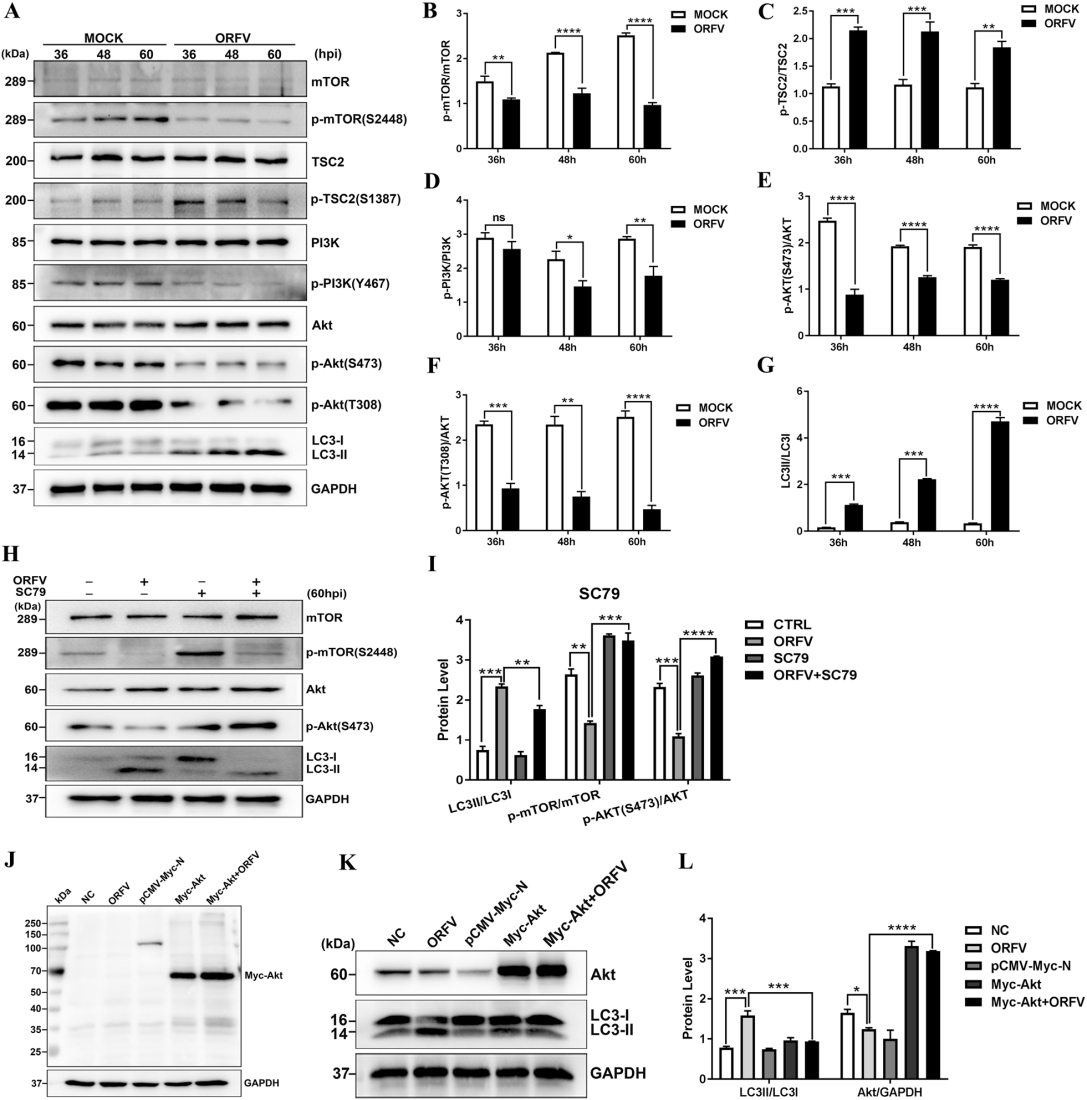
**ORFV induces autophagy by inhibiting PI3K/AKT/mTOR signalling. A** OFTu cells were mock infected or infected with ORFV-CL18. At 36, 48 and 60 hpi, the expression levels of p-mTOR (S2448), mTOR, p-TSC2 (S1387), TSC2, p-PI3K (Y467), PI3K, p-AKT (S473 and T308), AKT and LC3B were measured by Western blotting. GAPDH was used as the loading control. **B**–**G** The ratios of p-mTOR/mTOR **B**, p-TSC2/TSC2 **C**, p-PI3K/PI3K **D**, p-AKT (S473)/AKT **E**, p-AKT (T308)/AKT **F**, and LC3-II/LC3-I **G**. These protein bands were quantified by measuring the signal intensity with ImageJ, and the ratios are expressed as the mean ± SD (n = 3) (*P < 0.05, ** P < 0.01, *** P < 0.001, **** P < 0.0001; unpaired t test). **H** OFTu cells were mock infected, infected with ORFV-CL18, treated with 30 μM SC79 or treated with 30 μM SC79, infected with ORFV (MOI = 10) and incubated for 60 h. After treatment, the expression levels of p-mTOR (S2448), mTOR, p-AKT (S473), AKT and LC3B were measured by Western blotting. GAPDH was used as the loading control. **I** The ratios of p-mTOR/mTOR, p-AKT (S473)/AKT and LC3-II/LC3-I, which are expressed as the mean ± SD (*n* = 3) (**P < 0.01, *** P < 0.001, **** P < 0.0001; one-way ANOVA). **J** The recombinant vector pCMV-Myc-N-Akt was identified by Western blotting using an anti-Myc antibody. (K) OFTu cells were mock infected, infected with ORFV-CL18, transfected with pCMV-Myc-N or pCMV-Myc-N-Akt or transfected with pCMV-Myc-N-Akt, infected with ORFV (MOI = 10) and incubated for 60 h. After treatment, the expression levels of the autophagy marker proteins LC3B and Akt were measured by Western blotting. GAPDH was used as the loading control. **L** The ratios of LC3-II to LC3-I and Akt to GAPDH, which are expressed as the mean ± SD (*n* = 3). (*P < 0.05, *** P < 0.001, **** P < 0.0001; one-way ANOVA).

As mentioned above, ORFV induced autophagy by inhibiting mTOR, but the underlying mechanism of ORFV-induced autophagy remained unclear. PI3K/AKT/mTOR is a classical signalling pathway that regulates autophagy. We investigated the expression of PI3K and its downstream molecules related to the PI3K/AKT/mTOR pathway by Western blotting. As shown in Figures 4A–G, the phosphorylation levels of PI3K, as well as the phosphorylation levels of AKT at both the Ser473 and Thr308 sites, were reduced, the phosphorylation level of mTOR was reduced, and the LC3-II/LC3-I ratio was increased. Moreover, overexpression of AKT included by treatment with the specific AKT activator SC79 (Figures 4H, I) or transfection with an AKT overexpression vector (Figures 4K, L) inhibited the conversion of LC3-I to LC3-II. These results indicated that the PI3K/AKT/mTOR signalling pathway plays an important role in ORFV-induced autophagy.

### ORFV-CL18 induces autophagy by activating ERK1/2/mTOR signalling

**Figure 5:**
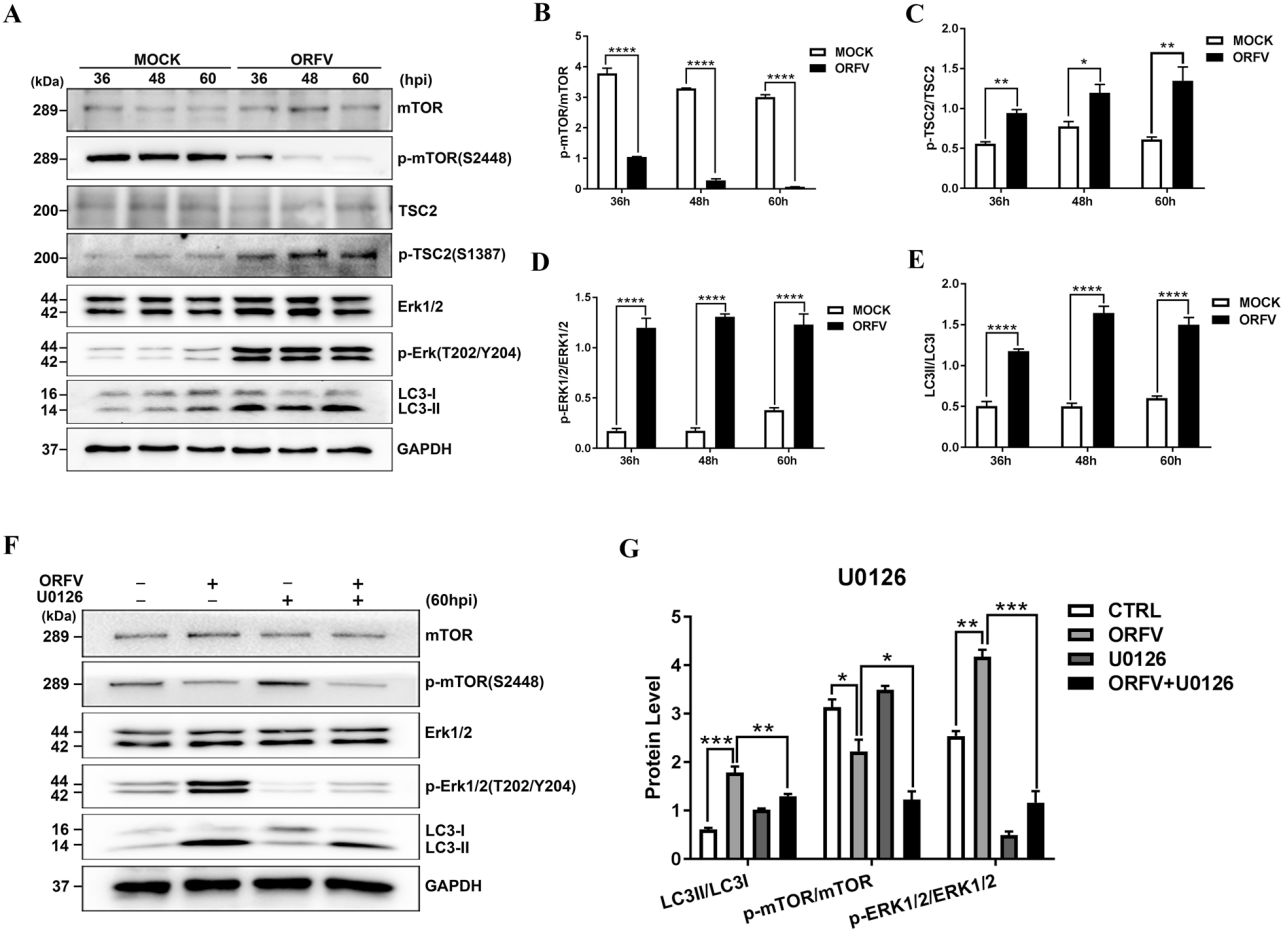
**ORFV induces autophagy by activating ERK1/2/mTOR signalling. A** OFTu cells were mock infected or infected with ORFV-CL18. At 36, 48 and 60 hpi, the expression levels of p-mTOR (S2448), mTOR, p-TSC2 (S1387), TSC2, p-ERK1/2 (T202/Y204), ERK1/2 and LC3B were measured by Western blotting. GAPDH was used as the loading control. **B**–**E** The ratios of p-mTOR/mTOR **B**, p-TSC2/TSC2 **C**, p-ERK/ERK **D** and LC3-II/LC3-I **E**. These protein bands were quantified by measuring the signal intensity using ImageJ, and the ratios are expressed as the mean ± SD (*n* = 3) (*P < 0.05, ** *P* < 0.01, **** P < 0.0001; unpaired t test). **F** OFTu cells were mock-infected, infected with ORFV-CL18, treated with 20 μM U0126 or treated with 20 μM U0126, infected with ORFV (MOI = 10) and incubated for 60 h. After treatment, the expression levels of p-mTOR (S2448), mTOR, p-ERK1/2 (T202/Y204), ERK1/2 and LC3B were measured by Western blotting. GAPDH was used as the loading control. **G** The ratios of p-mTOR/mTOR, p-ERK/ERK and LC3-II/LC3-I, which are expressed as the mean ± SD (*n* = 3) (*P < 0.05, ** P < 0.01, *** P < 0.001; one-way ANOVA).

Extracellular signal-regulated kinase 1/2 (ERK1/2) is another important upstream regulator of mTOR. A Western blot analysis showed that ERK1/2 was phosphorylated at both the Thr202 and Tyr204 sites, and the phosphorylation level of ERK1/2 was increased in ORFV-infected OFTu cells. Moreover, the phosphorylation level of mTOR was reduced, and the LC3-II/LC3-I ratio was increased in a corresponding fashion (Figures 5A–E). In contrast, treatment with the ERK1/2 inhibitor U0126 reduced the phosphorylation level of ERK1/2 and eliminated the inhibitory effect of ORFV on mTOR (Figures 5F, G). Therefore, we concluded that the ERK1/2-mTOR signalling pathway is involved in ORFV-activated autophagy.

Additionally, as an important energy-sensing kinase, AMPK is a crucial upstream target of autophagy. We therefore investigated the role of the AMPK signalling pathway in ORFV-activated autophagy. As shown in Additional file 1, ORFV induced autophagy by inhibiting mTOR signalling, while the expression levels of phosphorylated and unphosphorylated AMPK were not significantly changed (Additional files 1 A–E). Similarly, treatment with the AMPK activator AICAR (Additional files 1 F, G) or inhibitor Compound C (Additional files 1 H, I) did not change these levels. Thus, the AMPK signalling pathway may not be involved in the induction of autophagy by ORFV.

### Autophagy promotes ORFV replication in vitro

**Figure 6:**
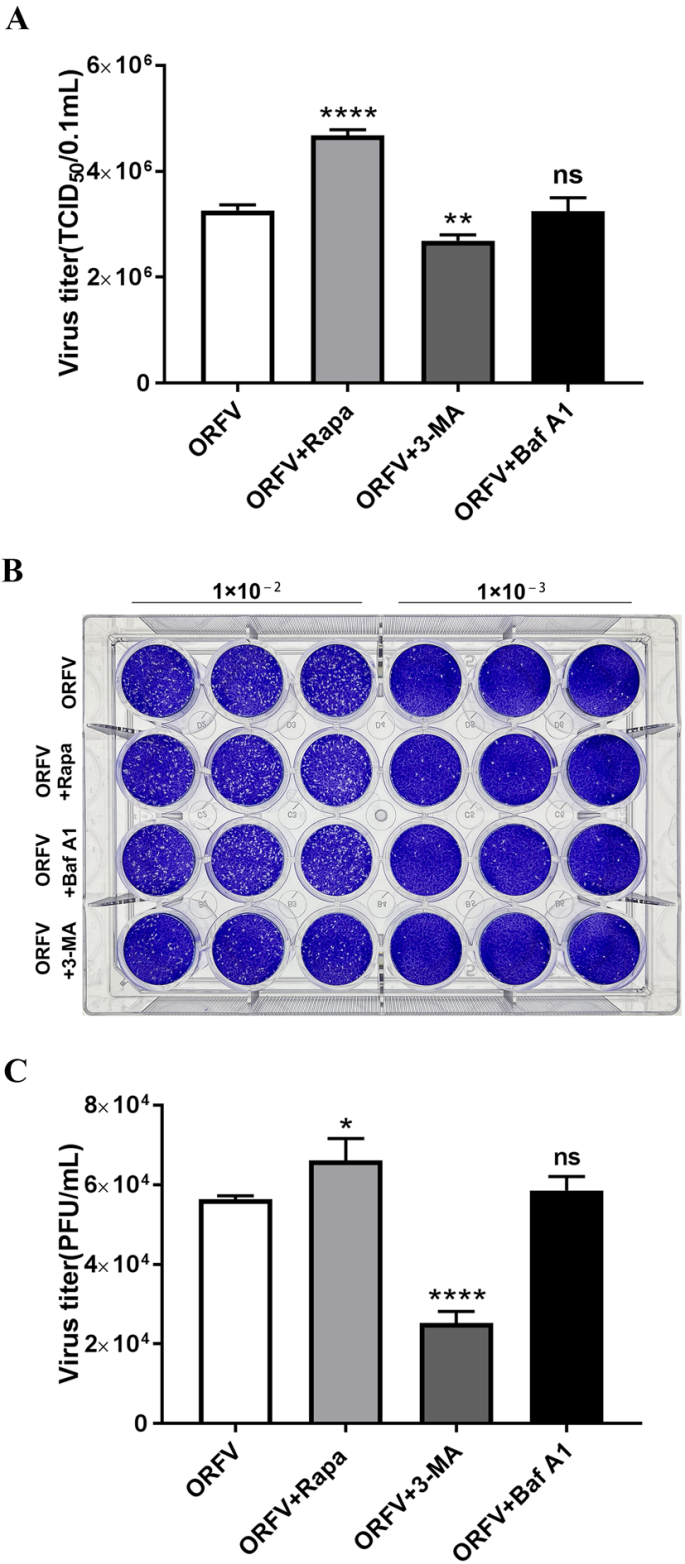
**ORFV titres are positively correlated with the level of autophagy. A** OFTu cells were treated with 30 μM Rapa, 5 mM 3-MA, 1 nM Baf A1 or normal medium and then infected with ORFV-CL18. At 60 hpi, the cell culture supernatant was harvested, and the virus titre was by TCID_50_ assay. The results are expressed as the mean ± SD (*n* = 3). One-way ANOVA was used to determine significance. Differences were considered statistically significant when *P* < 0.05. (**P < 0.01, **** P < 0.0001; one-way ANOVA). **B** OFTu cells were treated with 30 μM Rapa, 5 mM 3-MA, 1 nM Baf A1 or normal medium and then infected with ORFV-CL18 at different dilutions (1 × 10^–2^ or 1 × 10^–3^). At 60 hpi, the cell culture supernatant was harvested, and the virus titre was determined by the plaque assay. Each experiment was performed at least three times. **C** Plaques on the plate prepared with virus diluted 1 × 10^–2^ were counted, and the data are expressed as the mean ± SD (*n* = 3) (*P < 0.05, **** P < 0.0001; one-way ANOVA).

Autophagy plays vital roles in modulating viral replication and antiviral immune responses. To further explore the role of autophagy in ORFV proliferation, viruses under different autophagic conditions were harvested from ORFV-infected OFTu cells treated with rapamycin, 3-methyladenine and bafilomycin A1, and the virus titres were measured by TCID_50_ assay and plaque assay. The virus titres were higher in the rapamycin-treated cells than in the mock-treated cells. In the 3-methyladenine-treated cells, the virus titres were lower than those in the mock-treated cells. However, treatment with bafilomycin A1 exerted no significant effect on ORFV replication (Figures 6A–C). These results implied that ORFV-induced autophagy promotes its own replication.

### Pharmacological treatments do not affect cell viability

**Figure 7:**
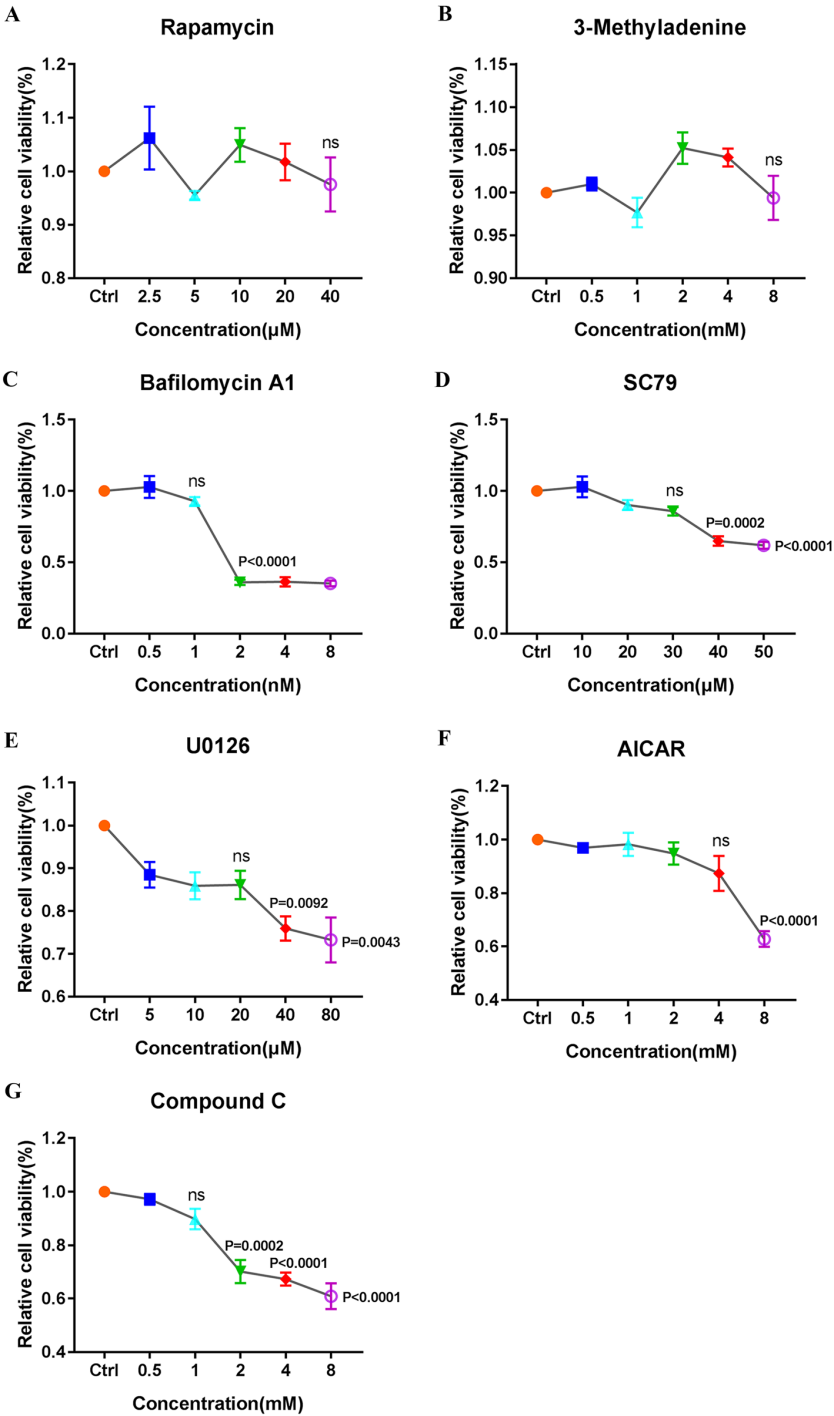
**Pharmacological treatments do not affect cell viability. A**–**G** OFTu cells were treated with Rapa (0, 2.5, 5, 10, 20 and 40 μM) (panel A), 3-MA (0, 0.5, 1, 2, 4 and 8 mM) (panel B), Baf A1 (0, 0.5, 1, 2, 4 and 8 nM) (panel C), SC79 (0, 10, 20, 30, 40 and 50 μM) (panel D), U0126 (0, 5, 10, 20, 40 and 80 μM) (panel E), AICAR (0, 0.5, 1, 2, 4 and 8 mM) (panel F) or Compound c (0, 0.5, 1, 2, 4 and 8 mM) (panel G). After treatment for 60 h, cell viability was determined by CCK8 assay. The percentage of viable cells is expressed as the mean ± SD. (*P < 0.05, ** P < 0.01, *** P < 0.001, **** P < 0.0001; one-way ANOVA).

To assess OFTu cell viability after treatment with rapamycin, 3-methyladenine, bafilomycin A1, SC79, U0126, AICAR or Compound C, a CCK8 assay was performed. As shown in Figures 7A–G, the concentration of the activator or inhibitor used in this study exerted no effect on cell viability.

## Discussion

Orf is an important zoonotic disease caused by ORFV. Due to an increasing number of hosts, ORFV has become an occupational hazard [25, 26]. Therefore, certain occupational groups, such as herders, veterinarians and butchers, are at higher risk of infection. In addition, farmers and zoo visitors may be infected when they are exposed to ORFV in nonoccupational environments due to scab shedding [27]. Notably, ORFV has caused serious economic losses in husbandry operation worldwide.

Autophagy is an essential component of host immunity and is leveraged by many viruses for survival. Recently, the interaction between autophagy and viruses has been widely investigated. Seneca Valley virus (SVV) [28], transmissible gastroenteritis virus (TGEV) [29] and other viruses with the ability to induce autophagy mediated through viral proteins. However, Middle East respiratory syndrome coronavirus (MERS-CoV) [30] and hepatitis C virus (HCV) [31] infection lead to autophagy blockage. Increasing evidence shows that autophagy may be a potential mechanism through which viruses establish successful infection, thereby playing a crucial role in many viral infections. Some viruses, such as influenza A virus (IAV) [32], porcine circovirus type 2 (PCV2) [33] and Muscovy duck reovirus (MDRV) [34], can leverage autophagy to potentially maximize their replication via interactions between viral proteins and autophagy-related proteins.

In our previous research, we found that ORFV infection induced autophagy in OFTu cells, but the exact molecular mechanisms underlying this autophagy induction remained unknown. The present study demonstrated that ORFV infection induced complete autophagy in OFTu cells. The autophagy protein LC3 was observed to undergo the transition from the cytoplasmic form (LC3-I) to the autophagosome membrane-bound form (LC3-II). The autophagy receptor P62 is a substrate for ubiquitination, is located on the autophagosome membrane, and can be encapsulated into the autophagosome for degradation by interacting with LC3 [35]. Thus, the expression level of P62 was measured by Western blotting, and a marked decrease in the number of ORFV-infected OFTu cells was observed. Considering that LC3 aggregates in the autophagosome membrane and is involved in the process of autophagosome assembly, the distribution of LC3 in autophagosomes was measured via confocal laser scanning microscopy. As shown in Figure 1H, GFP green fluorescent dot-like autophagosomes were also observed, which indicated that ORFV infection activated autophagy in OFTu cells. Autophagy is a dynamic process in which autophagosomes are constantly being formed and degraded; thus, the autophagic flux in ORFV-infected OFTu cells was measured after treatment with bafilomycin A1, an inhibitor of late-stage autophagy. Furthermore, autophagic flux was measured with an mRFP-GFP-LC3 expression vector, which was used to trace the formation of autolysosomes [36]. As shown in Figure 2C, GFP/RFP double-positive autophagosomes (yellow) were clearly observed in OFTu cells infected with ORFV 36 hpi and 48 hpi; nevertheless, RFP-positive autolysosomes (red) appeared in the cytoplasm 60 hpi and are considered markers of autolysosome formation. Moreover, typical double-membrane autophagosomes and single-membrane autolysosomes were observed via TEM, and the observations were consistent with the immunofluorescence results. Taken together, our results confirm that ORFV infection can induce complete autophagy and activate autophagic flux in OFTu cells.

In recent decades, autophagy has been reported to be regulated by different mechanisms and pathways. The canonical autophagy is regulated by mTOR and upstream AKT, MAPK and AMPK signalling pathways [8, 37, 38]. mTOR, a serine/threonine kinase, is the mammalian target of rapamycin protein that modulates autophagy and has been demonstrated to play an important role in autophagy regulation by controlling multiple sites of phosphorylation on ULK1, thereby affecting its translocation to phagophore assembly sites [39]. To determine whether ORFV induced autophagy through the mTOR signalling pathway, the expression of key molecules in the mTOR signalling pathway, including mTOR, TSC2, Rheb and P70S6K, was measured by Western blotting. The results indicated that ORFV infection activated autophagy by mediating the expression of upstream regulators (including TSC2, Rheb and P70S6K) of mTOR and thus inhibiting mTOR expression. Additionally, inhibition of mTOR expression using the chemical inhibitor rapamycin resulted in autophagy activation, but overexpression of Rheb led to autophagy blockage, which indicated that ORFV infection induces autophagy by inhibiting mTOR signalling pathway activation. To fully understand the precise mechanisms involved in ORFV-induced autophagy, the activation of the autophagy-specific PI3K/AKT/mTOR pathway in ORFV-infected OFTu cells was determined by Western blotting. Our results revealed that ORFV promotes autophagy in OFTu cells by inhibiting the phosphorylation of PI3K and AKT, subsequently resulting in Akt-mediated phosphorylation of TSC2, thereby allowing TSC2 to inhibit mTOR. Treatment with the Akt activator SC79 or transfection with an AKT overexpression vector inhibited ORFV-induced autophagy, further supporting the aforementioned conclusion. Furthermore, another known signalling pathway involved in the regulation of mTOR was investigated to determine the association between ORFV-induced autophagy and the MAPK/ERK1/2 signalling pathway. A series of conserved cascade signalling changes was observed to be accompanied by a significant increase in the phosphorylation of ERK1/2 and by mTOR inhibition, which demonstrated that the MAPK/ERK1/2/mTOR pathway is also involved in ORFV-induced autophagy. However, as illustrated in Additional file 1, our results revealed that activation or inhibition of ORFV-induced autophagy by AICAR or Compound C was independent of AMPK activity.

As an important component of innate immunity, autophagy plays an antiviral role by activating or enhancing the host immune response; in turn, autophagy can be inhibited or antagonized by viruses to enhance viral replication and infection [15, 40]. Furthermore, we aimed to reveal the effect of mTOR-mediated autophagy on ORFV replication in the presence of autophagy modulators. As shown in Figure 6, the pharmacological modulation of autophagy with rapamycin significantly increased virus yields (****P < 0.0001), while virus yields were significantly reduced in the 3-methyladenine treatment group (**P < 0.01) compared with the control group. However, we did not observe any significant alteration in the virus titre in bafilomycin A1-treated cells compared with control cells. Given these results, we concluded that mTOR-mediated autophagy can affect ORFV replication. In summary, ORFV induced complete autophagy by inhibiting the PI3K/AKT/mTOR signalling pathway and activating the ERK1/2/mTOR signalling pathway, and autophagy promoted ORFV replication in vitro. Notably, the result was not consistent with our previous observations in which autophagy did not seem to be essential for ORFV replication [41]. We speculated that the possible reason for the difference may been related to ORFV-CL18 [42], which was used in the study, might induce early stages of autophagy but block it at later stages, thereby increasing the titre of infectious ORFV. In addition, the higher multiplicity of infection (MOI) used in the study compared to that in the previous might have led to variations in the autophagic response.

In conclusion, our research extends our knowledge of the autophagy mechanisms induced by ORFV and presents a theoretical basis for the comprehensive analysis of the pathogenic mechanism of ORFV, showing great potential for its use in exploring new antiviral strategies.

## Supplementary Information

**Additional file 1: ORFV-induced autophagy shows no significant correlation with the AMPK/mTOR signalling pathway.** (A) OFTu cells were mock infected or infected with ORFV-CL18. At 36, 48 and 60 hpi, the expression levels of p-mTOR (S2448), mTOR, p-TSC2 (S1387), TSC2, p-AMPKα (T172), AMPK and LC3B were measured by Western blotting. GAPDH was used as the loading control. (B, C, D and E) The ratios of p-mTOR/mTOR (B), p-TSC2/TSC2 (C), p-AMPK/AMPK (D) and LC3-II/LC3-I (E). These protein bands were quantified by measuring signal intensity with ImageJ, and the ratios are expressed as the mean ± SD (*n* = 3) (*P < 0.05, ** P < 0.01, *** P < 0.001, **** P < 0.0001; unpaired t test). (F) OFTu cells were mock infected, infected with ORFV-CL18, treated with 3 mM AICAR or treated with 3 mM AICAR, infected with ORFV (MOI = 10) and incubated for 60 h. After treatment, the expression levels of p-mTOR (S2448), mTOR, p-AMPKα (T172), AMPK and LC3B were measured by Western blotting. GAPDH was used as the loading control. (G) The ratios of p-mTOR/mTOR, p-AMPK/AMPK and LC3-II/LC3-I, which are expressed as the mean ± SD (*n* = 3) (*P < 0.05, ** P < 0.01; one-way ANOVA). (H) OFTu cells were mock infected, infected with ORFV-CL18, treated with 1 mM Compound c or treated with 1 mM Compound c, infected with ORFV (MOI = 10) and incubated for 60 h. After treatment, the expression levels of p-mTOR (S2448), mTOR, p-AMPKα (T172), AMPK and LC3B were measured by Western blotting. GAPDH was used as the loading control. (I) The ratios of p-mTOR/mTOR, p-AMPK/AMPK and LC3-II/LC3-I, which are expressed as the mean ± SD (*n* = 3) (*P < 0.05, ** P < 0.01, *** P < 0.001, **** P < 0.0001; one-way ANOVA).
