# Supplementary figures and images for: Orf virus induces complete autophagy to promote viral replication via inhibition of AKT/mTOR and activation of the ERK1/2/mTOR signalling pathway in OFTu cells
Source: Vet Res. 2023 Mar 14;54:22. doi: 10.1186/s13567-023-01153-1 (PMC10013242; doi:10.1186/s13567-023-01153-1)

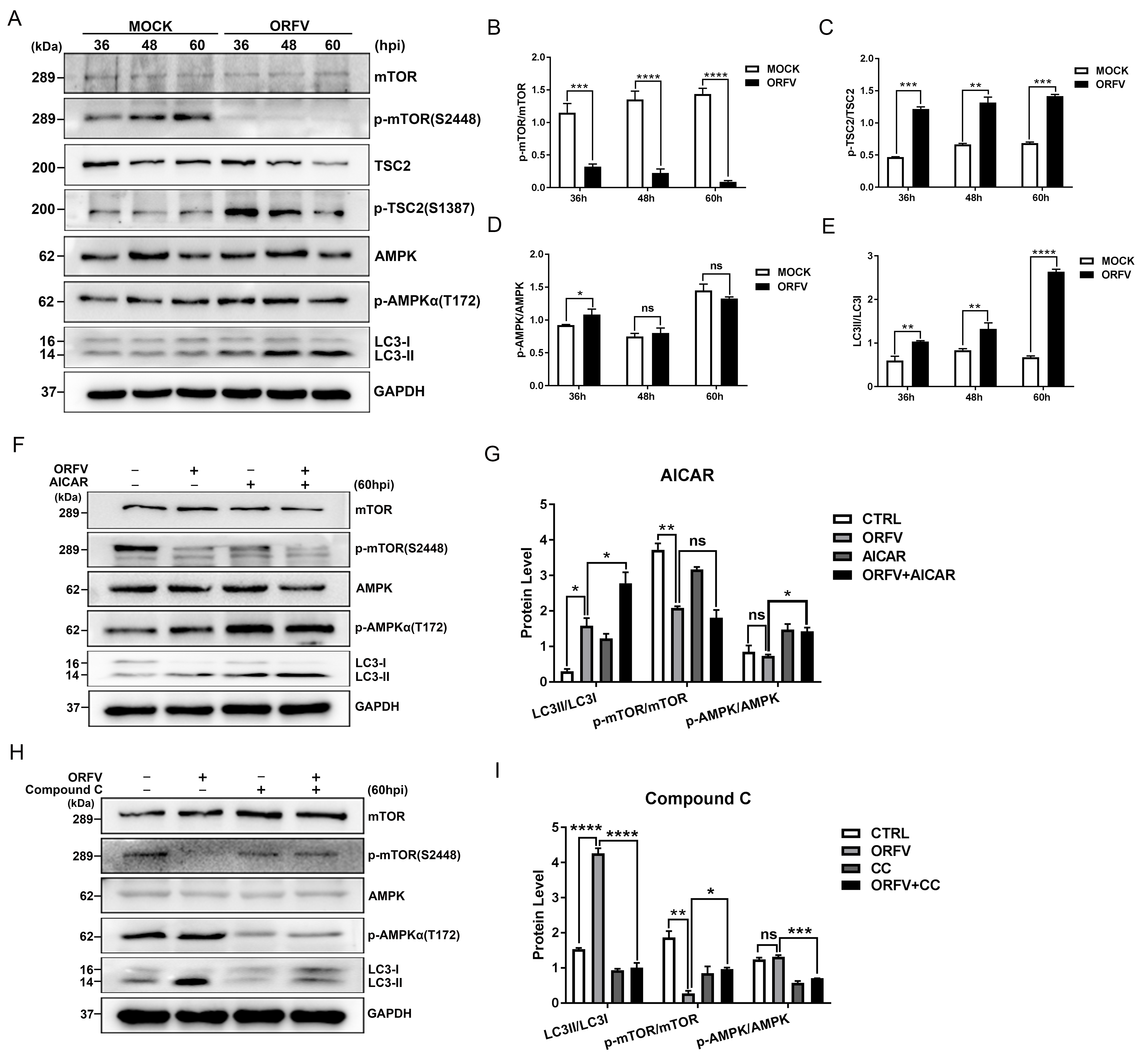

Supplement: Supplementary file 1 — Additional file 1: ORFV-induced autophagy shows no significant correlation with the AMPK/mTOR signalling pathway. (A) OFTu cells were mock infected or infected with ORFV-CL18. At 36, 48 and 60 hpi, the expression levels of p-mTOR (S2448), mTOR, p-TSC2 (S1387), TSC2, p-AMPKα (T172), AMPK and LC3B were measured by Western blotting. GAPDH was used as the loading control. (B, C, D and E) The ratios of p-mTOR/mTOR (B), p-TSC2/TSC2 (C), p-AMPK/AMPK (D) and LC3-II/LC3-I (E). These protein bands were quantified by measuring signal intensity with ImageJ, and the ratios are expressed as the mean ± SD (n = 3) (*P < 0.05, ** P < 0.01, *** P < 0.001, **** P < 0.0001; unpaired t test). (F) OFTu cells were mock infected, infected with ORFV-CL18, treated with 3 mM AICAR or treated with 3 mM AICAR, infected with ORFV (MOI = 10) and incubated for 60 h. After treatment, the expression levels of p-mTOR (S2448), mTOR, p-AMPKα (T172), AMPK and LC3B were measured by Western blotting. GAPDH was used as the loading control. (G) The ratios of p-mTOR/mTOR, p-AMPK/AMPK and LC3-II/LC3-I, which are expressed as the mean ± SD (n = 3) (*P < 0.05, ** P < 0.01; one-way ANOVA). (H) OFTu cells were mock infected, infected with ORFV-CL18, treated with 1 mM Compound c or treated with 1 mM Compound c, infected with ORFV (MOI = 10) and incubated for 60 h. After treatment, the expression levels of p-mTOR (S2448), mTOR, p-AMPKα (T172), AMPK and LC3B were measured by Western blotting. GAPDH was used as the loading control. (I) The ratios of p-mTOR/mTOR, p-AMPK/AMPK and LC3-II/LC3-I, which are expressed as the mean ± SD (n = 3) (*P < 0.05, ** P < 0.01, *** P < 0.001, **** P < 0.0001; one-way ANOVA). [file 13567_2023_1153_MOESM1_ESM.tif]
